# Direct Selection on Genetic Robustness Revealed in the Yeast Transcriptome

**DOI:** 10.1371/journal.pone.0000911

**Published:** 2007-09-19

**Authors:** Stephen R. Proulx, Sergey Nuzhdin, Daniel E. L. Promislow

**Affiliations:** 1 Department of Ecology, Evolution and Organismal Biology, Iowa State University, Ames, Iowa, United States of America; 2 Section of Evolution and Ecology, University of California at Davis, Davis, California, United States of America; 3 Molecular and Computational Biology, University of Southern California, Los Angeles, California, United States of America; 4 Department of Genetics, University of Georgia, Athens, Georgia, United States of America; University of California at Berkeley, United States of America

## Abstract

**Background:**

Evolutionary theory predicts that organisms should evolve the ability to produce high fitness phenotypes in the face of environmental disturbances (environmental robustness) or genetic mutations (genetic robustness). While several studies have uncovered mechanisms that lead to both environmental and genetic robustness, we have yet to understand why some components of the genome are more robust than others. According to evolutionary theory, environmental and genetic robustness will have different responses to selective forces. Selection on environmental robustness for a trait is expected to be strong and related to the fitness costs of altering that trait. In contrast to environmental robustness, selection on genetic robustness for a trait is expected to be largely independent of the fitness cost of altering the trait and instead should correlate with the standing genetic variation for the trait that can potentially be buffered. Several mechanisms that provide both environmental and genetic robustness have been described, and this correlation could be explained by direct selection on both forms of robustness (direct selection hypothesis), or through selection on environmental robustness and a correlated response in genetic robustness (congruence hypothesis).

**Methodology/Principal Findings:**

Using both published and novel data on gene expression in the yeast *Saccharomyces cerevisiae*, we find that genetic robustness is correlated with environmental robustness across the yeast genome as predicted by the congruence hypothesis. However, we also show that environmental robustness, but not genetic robustness, is related to per-gene fitness effects. In contrast, genetic robustness is significantly correlated with network position, suggesting that genetic robustness has been under direct selection.

**Conclusions/Significance:**

We observed a significant correlation between our measures of genetic and environmental robustness, in agreement with the congruence hypothesis. However, this correlation alone cannot explain the co-variance of genetic robustness with position in the protein interaction network. We therefore conclude that direct selection on robustness has played a role in the evolution of genetic robustness in the transcriptome.

## Introduction

Organisms are faced with the challenge of functioning and reproducing in the midst of both internal and external perturbations. Internal genetic changes arise due to mutation and recombination, while externally, organisms might experience a range of environments over various spatial and temporal scales, leading to variable selection pressures [1], [2]. How a species responds to these variable selection pressures depends on the details of the selective environment and the genetic variances and covariances for the traits under selection [3]. The genetic architecture of the traits of interest and the nature of intrinsic or extrinsic variability will, in turn, determine whether fitness is maximized by, on the one hand, plastically varying phenotype to match the external environment or internal genetic background, or on the other hand by producing a constant, robust phenotype [4], [5]. The idea that phenotypic insensitivity to the environment might be under selection was advanced by Waddington, Schmalhausen, and Thoday in the 1940s and 50s [6]–[9]. Of these early ideas, Waddington's concept of developmental canalization has perhaps incited both the most debate and the most theoretical modeling [10]–[12].

Theoretical models for the evolution of robustness have made clear the importance of distinguishing between environmental and genetic robustness (ER and GR, respectively) [10], [13]–[16]. We define environmental robustness as the insensitivity of a phenotype to environmental perturbations, while genetic robustness refers to the constancy of a phenotype when some component of the genotype is altered. In general, the strength of selection on robustness to some form of perturbation is limited by the fitness load that the perturbation can create [16]. However, theory suggests that the extent and causes of robustness evolution differ for ER versus GR. For environmental disturbances, selection on robustness depends on both the frequency and fitness cost associated with environmental changes [13], [16], [17]. Unlike environmental perturbations, the strength of selection on GR is not strongly related to the fitness cost associated with each genetic perturbation, but is instead related to the fraction of the overall mutational load that can be buffered by the focal gene [10], [13], [16]. This is because the mutation load is largely insensitive to the per-mutation fitness effect [18]. This leads us to predict that selection for genetic robustness will not be related to any measure of how important the trait of interest is to overall fitness. Rather, selection for genetic robustness is predicted to be related to the total frequency of deleterious effects that the focal gene can potentially buffer [16].

Given these theoretical results, we expect mutational robustness to be typically weakly selected while environmental robustness should be under strong selection [10], [13], [16], [19]–[22]. Several well known systems exhibit genetic robustness, but it is often linked to the mechanism of environmental robustness [23]–[26]. What, then, might account for the evolution of GR? Two main hypotheses have been proposed. The first, known as the congruence hypothesis, argues that genetic robustness has arisen as a byproduct of environmental robustness [10], [20], [25], [27], [28]. An alternative hypothesis, direct selection, argues that selection can act directly on both mutational and environmental robustness, and that selection has been strong enough to have an observable effect on mutational robustness in particular [10], [13], [16].

These two hypotheses lead to different sets of predictions. Under the congruence hypothesis, we expect that the traits that are most robust to environmental perturbations will also be those that are robust to genetic perturbations. This could occur, for instance, if enzymes that are selected to have excess capacity in order to produce consistent output under variable environmental conditions also turn out to have excess capacity in the face of genetic perturbations that reduce enzyme efficiency [27]. Furthermore, traits with high GR and ER will be those that are under the strongest selection.

**Figure 1:**
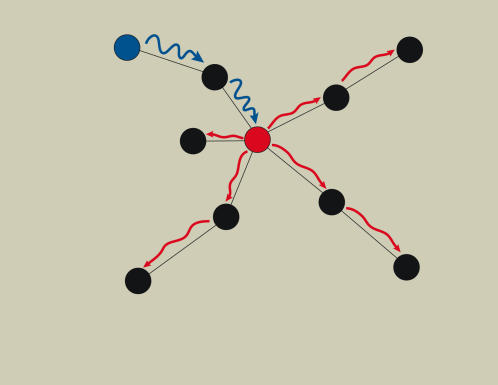
A schematic diagram illustrating the role that network position can play in propagation of noise through the network. The circles in the diagram represent genes while the lines represent bi-directional interactions (like protein-protein interactions). The diagram shows how a focal node, shown in red, can affect noise produced by a perturbed node, shown in blue. The noise produced by the blue gene is represented by the blue oscillating arrows, and is dampened after passing through the red gene. Because the red gene lies on pathways between many other genes it has a large potential to buffer genetic noise.

The direct selection hypothesis argues that natural selection can favor both ER and GR independently. However, the means by which this occurs differs for the two types of robustness. Traits that are strongly correlated with fitness are expected to evolve high ER. In contrast, traits with high GR will be those that have the greatest potential to buffer other traits. For example, if we measure robustness in levels of gene expression across genes in the genome, theory predicts that GR will be greatest for genes whose protein products have the potential to buffer the largest number of mutations. Proteins that interact with the largest number of other proteins in the protein-protein interaction network could potentially buffer mutations at each interacting locus, and are therefore predicted to be under stronger selection to create robustness (Figure 1). The direct selection hypothesis does not predict that ER will be strongly correlated with network structure, although we might expect to see some relationship due to the fact that highly connected proteins tend to be under stronger selection [29], though the correlation is relatively weak. The ability of a particular gene to buffer or amplify environmental variability is likely to depend on both direct interactions with the environment and indirect interactions, such as through signal transduction pathways. However, the fitness effects of altering expression at a focal gene could easily swamp out the effects of network position or, at a minimum, reduce the importance of network position. Thus, we have a distinct set of predictions that differentiate GR from ER, and congruence from direct selection.

While we have a clear body of theory and predictions, until now it has been difficult to obtain measures of genetic robustness, environmental robustness, selection intensity and network connectivity for a large number of traits in a single species. For example, detailed work on the segment polarity network of *Drosophila* has shown how genetic and environmental robustness are related in that specific network [26]. Induced mutation in *Drosophila* suggests that, for life history traits, both genetic and environmental robustness are correlated with the traits importance to fitness [20]. The structure of the chemotaxis network of bacteria has been shown to produce robustness to both genetic mutation and environmental perturbation [23]. Likewise, heat shock proteins have been shown to buffer both temperature effects and individual mutations in proteins that the chaperone interacts with [24]. However, each of these cases concerns robustness of a single trait. If we could study robustness at multiple traits simultaneously, we would have much more power to test theories of robustness. Datasets obtained from large-scale genomic studies now make this possible.

Here we use gene transcription data from the yeast, *Saccharomyces cerevisiae*, to test hypotheses for the evolution of robustness. By defining our traits of interest as the level of gene expression, a single organism provides us with measures of robustness for over 5000 traits (i.e., genes) simultaneously. While RNA production alone does not constitute a classical trait, it is an important step in producing functional proteins that contribute to adaptation. This gives us substantial power to examine the statistical relationships between robustness and potential causal variables using a common framework. For each trait, the phenotype within a given environment or genetic background is simply the level of gene expression relative to that of a control strain. Robustness for a single gene is defined as the relative constancy (i.e., the inverse of the variance) of that gene, measured across a series of environments or genotypes.

Our measures of ER and GR are derived from measures of variation in levels of gene expression across 15 different stressors in a total of 35 environments [ER, 30], and across a set of 167 non-lethal knockout mutations [GR, 31]. Gene knockouts are a particularly severe form of genetic perturbation, and are probably rare in nature. Cellular responses to such large perturbations could be different from responses to smaller-scale genetic changes. Accordingly, we also obtained measures of variation in gene expression across a set of 30 wild yeast strains collected from vineyards in California (genetic background robustness, or ‘BR’). These strains represent genomes that are capable of living and reproducing in the environment, and so represent a particularly interesting kind of genetic variation. These wild yeast strains were brought into the laboratory and then grown under identical conditions before RNA was extracted. Therefore, expression levels measured for each gene show their response to a change in the genetic background.

We found that these three forms of robustness were significantly correlated at a genomic scale. However, environmental and genetic robustness differ in their relationships to putative causal variables that represent both the evolutionary history and network position of genes. These causal variables include essentiality (whether a gene knockout is lethal or not), evidence of purifying selection as measured by *K_a_*/*K_s_* ratios, and knockout growth effects, which we assume to be measures of ‘gene importance’. We also include several measures of the structure of protein networks and gene regulatory networks [5], [29], [32]. Environmental robustness was significantly correlated with several measures of gene importance, while genetic and background genotype robustness were correlated with position in the protein network. While we cannot rule out the possibility that congruence explains a portion of the observed genetic robustness, our analysis provides evidence that direct selection has a measurable effect on genetic robustness.

## Results

### Correlations between traits

**Figure 2:**
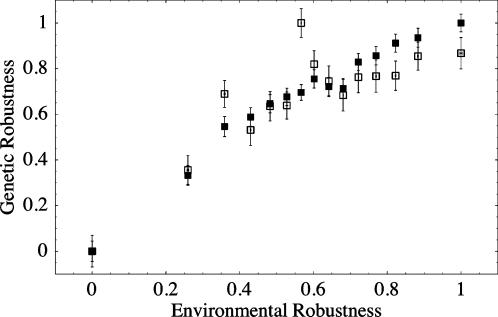
The relationship between environmental expression robustness and two forms of genetic robustness. The data were separated into 15 bins based on ranked environmental robustness. For each bin the mean and standard error of each form of robustness was calculated. Filled squares indicate robustness to knockouts while open squares indicate robustness to background genotype. All statistical analyses were carried out on the un-binned data.

We found that environmental robustness was positively and significantly correlated with both measures of genetic robustness (GR and BR) (figure 2), and that all three measures of robustness were positively correlated with one another (Spearman's *ρ*, ER-GR: *ρ* = 0.26, ER-BR: *ρ* = 0.13, GR-BR: *ρ* = 0.18; *P*<10^−20^ in all cases). In addition to the non-parametric correlations, we used linear regression to determine the correlations between each of the three measures of robustness. Each of the three possible pairwise regressions were highly significant with p<10^−20^.

**Figure 3:**
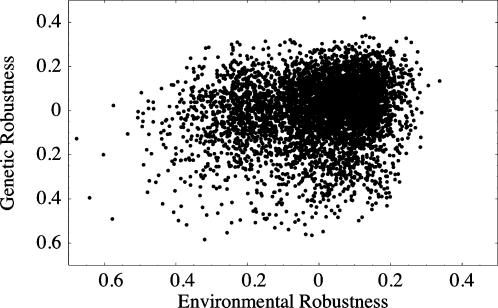
Correlation between residual GR and residual BR. We independently fit GR and BR to ER and performed a linear regression. Residual values of GR and BR were calculated and are shown here. The measures are significantly correlated with Spearman's *ρ* of 0.15 and a Pearson's r of 0.16.

The congruence hypothesis posits that both GR and BR are byproducts of selection on ER. If GR and BR had evolved solely in response to ER, we would expect that GR and BR would no longer be correlated after removing the effects of ER through partial regression. Put another way, under the direct selection hypothesis, we expect a correlation between GR and BR even after controlling for the statistical effects of ER. The two measures of genetic robustness are, in fact, correlated with one another after removing the effects of ER. We also used both non-parametric and regression based approaches to assess the partial correlation between GR and BR. We performed a multiple regression with ER and BR as factors and GR as the response variable. The model was highly significant with p<10^−30^ and had partial regression coefficients that were significantly positive. In particular, the two measures of genetic robustness were positively related (β = 0.16, s.e. = 0.014). Further, the multiple regression explained 8.6% of the total variance. We also took another, partially non-parametric approach to calculate the correlation between GR and BR. We computed residual GR and BR from a linear regression against ER. Their residual values are highly correlated with a coefficient similar to that of their raw values (figure 3; Spearman's *ρ* = 0.15, p<0.0001).

The direct selection hypothesis predicts that only the environmental robustness of a trait should be correlated with the intensity of selection acting on that trait, while the congruence hypothesis predicts that both ER and GR should be correlated with the intensity of selection. We used three measures of gene importance as proxies for the intensity of selection; whether a gene was lethal when knocked out, the *K_a_/K_s_* ratio and whether colony growth was affected by heterozygous gene knock-outs. We found that all three types of robustness were higher in genes that are lethal when knocked out (Table 1). However, the other measures of gene importance were correlated with ER but not with GR or BR. While the lethality data suggested that genes that are more important determinants of fitness have greater environmental robustness, our analysis of *K_a_/K_s_* showed the opposite pattern. Genes that historically have experienced the strongest intensity of selection (i.e., low *K_a_/K_s_* values) showed the *least* robustness. The relationship between the effect of a heterozygous gene knockout on growth rate and ER depended on the medium. Genes that reduce growth in complete media are associated with lower robustness while genes that reduce growth in minimal media are associated with higher robustness.

**Table 1:** Predictors of robustness (correlation coefficient, ±s.e.).

	Log *Ka/Ks*	Lethality	Het KO Complete	Het KO Minimal
ER	**0.081 (0.0073)**	**0.011 (0.0033)**	**0.38 (0.16)**	***−0.16 (0.062)***
GR	0.011 (0.0077)	**0.023 (0.0035)**	0.0039 (0.17)	−0.041 (0.065)
BR	−0.014 (0.0078)	**0.017 (0.0035)**	0.18 (0.17)	−0.0047 (0.067)

We analyzed a multiple regression model with *K_a_/K_s_* ratio, lethality, and colony growth of heterozygous knockouts in complete and minimal media as potential predictors of robustness. Bold numbers indicate P<0.01, bold italics P<0.05. For ER, *K_a_/K_s_* ratio, lethality, and heterozygous knockout growth rates, each are significant predictors of robustness. In contrast, lethality was the only measure of gene importance that was significantly correlated with GR and BR.

We also tested whether genes that have environment specific growth responses show a relationship with expression robustness. We defined a gene as having a differential growth effect if a heterozygous knockout mutant caused lower growth in one media (YPD or minimal) but not the other [32]. We found a significant relationship between ER and differential growth, with differential growth associated with low robustness (F = 12.76, DF = 1, p<0.001). In contrast, GR and BR were not significantly associated with differential growth (GR F = 1.24, DF = 1, p = 0.26; BR F = 0.0024, DF = 1, p = 0.96).

Under the congruence hypothesis, GR and BR evolve as byproducts of ER and would therefore not be expected to have statistical relationships with gene importance once the effects of ER were statistically controlled for. We calculated the residual values of GR and BR from linear regressions against ER and used those residual robustness values to test for effects on each of our measures of gene importance. Lethal genes were associated with higher residual robustness. In contrast, high *K_a_/K_s_* was associated with lower residual robustness. This indicates that genes under stronger purifying selection are more genetically robust than expected, given their level of ER (GR: F = 6.61, p = 0.01, slope = −0.018 (0.0076); BR: F = 17.89, p<0.0001, slope = −0.031 (0.0074) ). Colony growth and differential colony growth were not significantly associated with either measure of residual genetic robustness.

### Network structure

The direct selection hypothesis for the evolution of robustness predicts that genetic, but not environmental, robustness should be correlated with the ability of a gene to buffer changes in a network of interacting genes. Of the three measures of network centrality (degree, closeness and betweenness), ER was positively correlated with degree but not with closeness or betweenness (table 2). In contrast, both GR and BR were correlated with all three measures of network centrality (*P*<10^−7^ in all cases). More central and higher degree proteins had higher robustness than less central proteins. After adjusting for multiple comparisons using a Bonferroni adjusted critical α = 0.0056, however, only GR and BR were significantly correlated with any measure of network position.

**Table 2:** Spearman's *ρ* correlation between measures of robustness and protein centrality.

	ER	GR	BR
Degree	***0.037***	**0.097**	**0.11**
Closeness	0.0034	**0.067**	**0.087**
Betweenness	0.027	**0.0792**	**0.086**

We calculated the ranked correlation using all genes, regardless of whether they were in the central component or not. This means that unconnected genes were assigned a closeness and betweenness of 0. Numbers in bold have *P*<0.0001 while bold italics indicate *P*<0.05.

The number of genes that regulate a focal gene is known to be positively correlated with ER [5]. The number of regulators was also positively correlated with both GR and BR. Position in the protein network is also correlated with position in the regulatory network, and this could produce the observed relationship between robustness and protein centrality. Because the number of regulatory factors has been previously shown to effect expression robustness [5], we wanted to ensure that these observed patterns were not simply caused by correlations with the number of regulatory binding sites (and putative regulators) at a gene (K_in_). All measures of robustness were negatively correlated with Log-transformed values of K_in_ (ER *β*  = −0.070±0.013, GR *β* = −0.11±0.014, BR *β* = −0.037±0.013). In order to statistically correct for the effect of K_in_ we first performed linear regression with of each measure of robustness against Log K_in_ and calculated the residual robustness. We then measured the correlation between residual robustness and network position and found that more central proteins had higher residual GR and BR (*P*<0.00001 for degree, *P*<0.05 for closeness and betweenness). No measure of centrality was correlated with residual ER.

### Relationship to Protein Variability

We obtained data from [33] on cell-to-cell variation in protein abundance. These data were collected from a library of fluorescently tagged yeast strains in a constant environment. Data were available both for yeast raised in complete (YPD) and minimal media. We first calculated the residual of the log variance in protein abundance from a cubic spline fit (λ = 0.1) with log protein abundance (LRV). This allowed us to remove the effect of protein abundance *per se* and determine if proteins with noisier expression were associated with robustness. We calculated correlations between our measures of robustness and LRV using both Spearman's *ρ* and Pearson's r. Table 3 shows that all measures of robustness are always significantly negatively associated with protein variability. We performed a multiple regression with LRV and K_in_ as factors and found that, for each measure of robustness, LRV still had a significant effect and that this effect was in the same direction as the pairwise correlation (Table 4). Thus, genes that had relatively high levels of variation in expression between environments and genetic backgrounds also had relatively high levels of variation in protein abundance within a single environment.

**Table 3:** Correlations between robustness and log residual protein abundance (LRV).

	LRV, Complete				LRV, Minimal			
	*&SetFont Typeface="12";ρ*	p	R	p	*&SetFont Typeface="12";ρ*	P	R	p
ER	−0.11	1.9E-7	−0.22	1.2E-26	−0.12	1.5E-7	−0.19	1.9E-18
GR	−0.20	4.9E-22	−0.26	4.2E-35	−0.19	1.4E-17	−0.22	2.2E-24
BR	−0.12	3.9E-9	−0.13	3.3E-9	−0.061	0.0058	−0.10	3.0E-6

Proteins that have low variability for their expression levels tend to have high robustness.

**Table 4:** Multiple regression results for robustness as predicted by log protein abundance (LRV) and K_in_.

	K_in_	p	LRV, Complete	p
ER	−0.064	0.00080	−0.32	3.1E-11
GR	−0.091	1.8E-6	−0.38	2.0E-14
BR	−0.027	0.15	−0.19	1.0E-4

Genes with larger numbers of binding sites and increased protein variability have lower robustness.

## Discussion

Two competing hypotheses—direct selection and congruence—have been put forward to explain why some traits might be more robust than others when faced with environmental or genetic perturbations. Our study of variation in transcription levels in the yeast genome provides us with the first large-scale and simultaneous test of these two hypotheses.

Our results provide support, albeit mixed, for both hypotheses. In line with the congruence hypothesis, all three measures of robustness (ER, GR and BR) were correlated with one another. However, if congruence alone were responsible for the evolution of GR and BR, then we would not expect GR and BR to be independently correlated with other causal variables. Since we find that GR and BR are correlated with each other once the effects of ER are statistically removed, that GR and BR are not correlated with *K_a_/K_s_* or growth effects, and that GR and BR are each correlated with network position, we conclude that there has been direct selection for genetic robustness.

We measured several traits associated with the relative importance to fitness of each gene. The direct selection hypothesis predicts that only ER should be correlated with measures of gene importance. In fact, we found that ER was significantly correlated with each measure, whereas GR and BR were only correlated with lethality. On the face of it, this would appear to support the direct selection hypothesis. However, one result was unexpected, and in direct contrast to our prediction. The direct selection hypothesis predicts that robustness should be greatest in genes that exhibit the highest measures of selection intensity. In fact, we found just the opposite. Genes with low *K_a_*/*K_s_* ratios and genes that lowered growth rate when knocked out in minimal media had low environmental robustness. We return to this result later in the discussion.

Further supporting the direct selection hypothesis, GR and BR were more strongly correlated than was ER with measures of network centrality. This is in line with the theoretical prediction that genes that are better able to buffer genetic mutations at other loci will evolve higher genetic robustness. Our results show that more central proteins have higher GR and BR, indicating that highly connected genes maintain relatively constant levels of mRNA expression under a range of genetic perturbations. This would lead to robustness in terms of biological function if the presence of these robustly expressed proteins could buffer or compensate for changes in abundance or sequence of other proteins with which they interact.

Perhaps the most surprising result was that genes with high environmental robustness appeared to be under less intense selection than those that varied more across environments. This may seem to indicate that natural selection is favoring a lack of robustness. However, an alternative interpretation of these results is possible if we think of high variability across environments not as low robustness, but rather as high, and potentially adaptive, phenotypic plasticity. Phenotypic plasticity can evolve as an adaptive response to variation in the environment when selection favors alternative phenotypes in different environments [4], [5]. Thus, our observation that genes under strong stabilizing selection also have low ER is consistent with the evolution of adaptive plasticity in expression levels.

The observation that low *K_a_*/*K_s_* was associated with low ER suggested that our analysis of heterozygous knock-outs may have been incomplete. If the optimal level of expression of a gene were environment specific, then we would expect that that gene would have environment specific effects on fitness when its expression was artificially altered. To this end, we defined a gene as having a differential growth effect if a heterozygous knockout mutant caused lower growth in one growth media but not the other. Genes that have differential growth effects are likely to have different optimal expression levels in different environments, because the experimental protocol manipulates expression level in an environment independent way. We found that low ER was associated with differential growth effects, suggesting that genes that are highly variable in expression with respect to environment are likely to have environment specific fitness consequences when perturbed.

Previous work has suggested that plasticity in expression is functionally related to the number of transcription factors that regulate a focal gene [5]. Because transcription factor genes are themselves responsive to changes in environmental conditions, it is not surprising that genes with more regulatory inputs have increased environmental plasticity. We found that genetic plasticity also increased with the number of regulatory inputs, although to a lesser degree than for environmental plasticity. On the other hand, genes that are more connected and more central in the protein interaction network have increased expression robustness to genetic perturbations even when we controlled for the number of regulatory inputs.

Further evidence regarding the congruence hypothesis is provided by analyzing the residual levels of GR and BR from a regression with ER. If GR and BR had evolved solely as a by-product of ER, then we would also expect that their residual values would have no relationship with lethality, purifying selection, or differential growth. Surprisingly, high residual GR and BR was associated with low *K_a_/K_s_*, opposite to the pattern that we saw with raw ER and *K_a_/K_s_*. Because genes with lower *K_a_/K_s_* values had higher residual genetic robustness, we can infer that there is weak canalizing selection on expression robustness to genetic perturbations, but that this is often overwhelmed by selection for environmental plasticity. This implies that observed robustness represents a balance between congruence acting to allow transcription responses to environmental change and direct selection of genetic robustness to remain insensitive to genetic perturbations.

We were also interested in determining if within-environment variability in expression explained our robustness data. We examined the correlation between robustness and protein variability among cells as measured by [33]. We found that relative variance in protein expression, when the effects of protein abundance were removed, was negatively correlated with robustness, indicating that genes with greater robustness had relatively lower cell-to-cell variability in protein abundance. One possible explanation for this relationship is that genes that have larger numbers of regulators are more responsive to changes, both because of stochastic variation in a constant environment and in response to larger magnitude environmental and genetic changes [5]. However, a multiple regression with the number of regulators and protein variability as factors predicting both forms of robustness showed an independent effect of protein variability on robustness. This surprising result reinforces our conclusion that variability in gene expression represents the outcome of evolutionary pressures to maintain robust expression under some situations while allowing plastic expression under others.

While we found a highly significant correlation between network position and genetic robustness, the rank correlation coefficients were in the range of 0.05 to 0.11, leaving much variation in robustness unexplained. Our measures of network position were relatively crude and required separate analysis of information from the protein network and transcription network. An important future goal is to develop a better understanding of the topological features of gene networks that make them vulnerable to damage [34]. This approach is likely to provide additional insight into the complex relationships between genetic and environmental robustness.

### Final Thoughts

We used whole genome expression data to evaluate two competing hypotheses for the evolution of expression robustness. The congruence hypothesis posits that selection for environmental robustness leads to the evolution of genetic robustness as a correlated response. In contrast, the direct selection hypothesis posits that selection acts independently on environmental and genetic robustness. In their strictest interpretation, these hypotheses have different predictions. However, direct selection on both forms of robustness could potentially create unexpected auxiliary correlations. For instance, if the same sets of genes were under selection to become both more environmentally and more genetically robust, then we might expect correlations between our measures of environmental and genetic robustness even without correlated evolution. Likewise, since measures of gene importance are often correlated with network position, direct selection on ER might produce a correlation between ER and network position. These effects would only cloud our analysis if the correlation between gene importance and network position were tight, but regressions of protein degree against lethality, *K_a_/K_s_*, and heterozygous knockout growth rate have R^2^ values less than seven percent. In addition, we did find significant relationships between measures of genetic robustness and measures of network position that could not be explained by congruence alone.

While we cannot entirely rule out the possibility that direct selection on both forms of robustness is responsible for all of our results, we can rule out the possibility that congruence alone explains the observed pattern of genetic robustness. First, we observed a strong correlation between GR and BR even when the effect of ER was statistically controlled for. For this to be explained by the congruence hypothesis it would need to be the case that the correlated responses of both GR and BR with ER had a common mechanistic basis. Second, GR and BR did not show the same pattern of correlation as ER with our measures of gene importance. This suggests that, to the extent that correlated evolution plays a role in genetic robustness evolution, it is not any more intense when direct selection is acting on ER. Third and most significantly, GR and BR showed tighter correlations with measures of protein network centrality than did ER, and this pattern is only predicted by the direct selection hypothesis. Further, when we statistically controlled for the effect of the number of regulatory inputs on expression robustness, only GR and BR were significantly correlated with network position.

Taken together then, our results show a clear signature of direct selection acting on genetic robustness and further suggest that similar forces act on robustness to knockouts and robustness to changes in the genetic background. While we observe a correlation between our measures of environmental robustness and genetic robustness, the pattern of correlation between the putative causal variables (gene importance and network position) and each form of robustness is intriguing. In particular, while all measures of gene importance were correlated with environmental robustness, only lethality was correlated with genetic robustness. Conversely, all measures of network centrality were strongly correlated with GR and BR, but only network degree was correlated with environmental robustness, and the correlation was weak. Finally, there was no significant correlation between *K_a_*/*K_s_* and absolute measures of GR, but for a given value of ER, genes that have higher GR are under stronger purifying selection.

How can we understand this suite of results? One possible explanation is that environmental and genetic robustness share a common mechanistic basis, but the direction of selection acting on these two traits may be divergent. Thus, genes under weaker selection for robustness would contribute to a positive correlation between environmental and genetic robustness. Genes under strong and divergent selection for robustness would reveal distinct correlations between each form of robustness and variables that are related to selection on robustness. For expression robustness, variation in the number and type of transcription regulators could be a mechanism that affects both expression robustness and adaptive plasticity [5]. For instance, when a gene acquires additional regulatory elements that allow it to respond adaptively to environmental change, it may necessarily make expression of that gene sensitive to genetic changes at other loci, a trade-off predicted by recent complex systems theory [35]. If this trade-off is an unavoidable feature of gene expression networks, then it may well be that one cost of phenotypic plasticity is a reduction in genetic robustness.

## Methods

### Expression Data

We analyzed expression for the yeast *Saccharomyces cerevisiae* that had been exposed to environmental perturbations (ER), gene knockouts (GR), and changes in genetic background (BR). We measured the expression variability by calculating the mean and variance of expression changes of each gene in the yeast genome in response to each form of perturbation. In all datasets analyzed here, we find a strong mean-variance correlation—genes with large changes in expression levels relative to controls also show increased variance. We controlled for the possible bias created by the mean-variance correlation by calculating the residual of variance in expression versus mean expression using a cubic spline fit (λ = 0.1). Thus, the corrected measure of variance for the *i*th gene is Vc_i_. We calculated robustness as the relative invariance of expression by linearly transforming each measure of residual variance so that values with the lowest variance had a robustness value of 1 while values with the highest variance had robustness values of 0. Thus, the robustness of the *i*th gene is given by
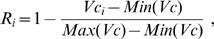
where the minimum and maximum functions are taken over all genes in our sample. We independently performed this transformation on each of our gene expression datasets to obtain ER, GR, and BR.

Data on gene expression were obtained from Gasch *et al*. [30] and Hughes *et al*. [31]. Additional experiments, described below, were performed by S. Nuzhdin using wild yeast strains from the UC Davis collection. Gasch *et al*. and Hughes *et al*. reported expression ratios of unique mRNA sequences in response to genetic and environmental perturbations as compared to a wild-type strain. The Gasch *et al*. dataset included 6,152 genes while the Hughes *et al*. dataset included 6,287 genes. We limited our analysis to genes that correspond to known proteins as listed in the Comprehensive Yeast Genome Database (http://mips.gsf.de/genre/proj/yeast/)[36], reducing our dataset to 5266 genes.

To calculate ER, we used the Gasch data on expression ratios for the set of environmental perturbations to calculate the mean expression ratio and the variance in the expression ratio for each gene. This included 167 experiments with 15 specific stressors, including heat shock, hyper- and hypo-osmolarity, and a number of chemical exposure treatments. We selected a subset of environmental perturbations from the dataset consisting of 35 experiments and 15 stressors. These experiments were selected to minimize pseudoreplication of similar environmental perturbations. For example, only one experiment that involved a temperature shift from 37°C. to 25°C. was retained out of five such experiments. The dataset that we used can be obtained from http://www-genome.stanford.edu/yeast_stress/data/rawdata/complete_dataset.txt. The experiments that we used correspond to columns 11, 18, 20, 24, 35, 38, 48, 57, 65, 70, 80, 87, 92, 93, 96, 101, 108, 110, 116, 125, 126, 137, 151, 152, 153, 154, 155, 156, 157, 158, 159, 160, 161, 162, 163. Descriptions of these experiments can be found at http://www-genome.stanford.edu/yeast_stress/materials.pdf [30].

To calculate GR we used the Hughes *et al*. dataset, which included 276 unique gene knockout experiments. We calculated the mean expression ratio and the variance in the expression ratio over all experiments. We calculated BR using 30 wild yeast strains of *S. cerevisiae*.

If we find that genes differ in how variable their expression levels are across genotypes or environments, this difference could be due to intrinsic differences in variability among genes, and not to their response to environmental or genetic perturbations. Accordingly, we also compared our measures of robustness with intrinsic measures of variability for protein levels within a constant environment, using data from [33]. We tested for rank correlations between each of our measures of expression robustness and the coefficient of variation for protein expression (CV) measured in both complete and minimal media.

### Expression analysis of wild *S. cerevisiae* stocks

The genetic background data (GR) were collected from 30 stocks from the UC Davis collection of natural *S. cerevisiae* (kindly provided by Linda Bisson, UC Davis). Microarrays were performed between overlapping pairs of strains in a dye-swapped design. Data were normalized to correct for dye effects using Agilent software. The expression of each gene was inferred using an ANOVA technique to estimate the expression level of each gene in each strain [37]. This allowed us to estimate the effect of each genetic background on the expression of each gene and calculate the variance in log expression over all strains (See supplemental Data S1). We also calculated the mean expression level of each gene. Because these analyses did not include a single control wild-type strain to generate log expression ratios, the mean expression across all strains was used as the baseline expression value for each gene.

### Gene Importance

We used three approaches to determine how important each gene is to fitness in a yeast cell. First, genes were classified as viable if a knockout mutant could grow and survive. Viability data were obtained from the Comprehensive Yeast Genome Database [36]. Second, we obtained data on the historical strength of purifying selection as measured by *K_a_/K_s_* ratios, estimated in reference [38] (data available at ftp://ftp-genome.wi.mit.edu/pub/annotation/fungi/comp_yeasts/S4.MutationCounts/b.KaKs_details.xls] . Because there is a significant relationship between *K_a_/K_s_* ratio and viability (d.f. = 2746, *t* = 7.93, p<0.0001) we performed ANCOVA with viability as a fixed effect and log_10_ *K_a_/K_s_* as the covariate. Third, we used data from [32] to determine which genes had significant effects on colony growth when their expression levels were altered, assuming that heterozygous knockouts have reduced expression. We used measurements of the reduced growth rate of heterozygous knockout lines in both complete and minimal media. We used a linear model with the four measures of gene importance as causal variables to test for effects on each form of robustness.

### Network Position

We measured network statistics of genes in both the yeast gene regulatory network [39] and the yeast protein-protein interaction network [40]. The regulatory network is a directional network with a small number of regulators (94 genes used in this study) and a larger number of regulated genes (1482 genes used in this study, 31 of which were also regulators). We calculated both the out degree, K_out_ (number of genes regulated by the focal gene) and the in degree, K_in_. We used the dataset from Lee et al. to find predicted regulatory interactions at the *P* = 0.001 level.

Data on physical protein interactions were obtained from Yeast Grid (http://biodata.mshri.on.ca/yeast_grid/servlet/SearchPage) [41]. We excluded data based on synthetic lethal and dosage lethal interactions because they are not necessarily based on physical interactions and have not been systematically determined. Our network contained 4,692 genes involved in 15,035 interactions [40], [42]–[48]. We used Pajek [49] to calculate the degree, betweenness, and closeness of all genes in the network.

### Statistical Methods

All statistical calculations were performed using JMP 5.1.2 (SAS Institute). We calculated the Spearman's *ρ* statistic to determine non-parametric correlations of untransformed data. For regressions and all other parametric tests we used transformed data to minimize deviations from normality. Expression variance was log transformed before calculating the cubic spline residuals. Log_10_ transformations were also performed on *K_a_/K_s_* ratios, K_in_, and K_out_.

## Supporting Information

Data S1 Wild yeast strain expression data.
(3.66 MB XLS)
